# The Relationship between Role Ambiguity and Workers’ Creativity during the COVID-19 Pandemic in China

**DOI:** 10.3390/ijerph192315977

**Published:** 2022-11-30

**Authors:** Jing Zhang, Yidan Hong, Andrew P. Smith

**Affiliations:** 1 School of Humanity and Law, Social Governance Innovation Research Center, Henan Agricultural University, Zhengzhou 450046, China; 2 Mental Health Center, Hainan College of Foreign Studies, Wenchang 571321, China; 3 Centre for Occupational and Health Psychology, School of Psychology, Cardiff University, Cardiff CF10 3AS, UK

**Keywords:** role ambiguity, work-related rumination, creativity, Demands–Resources–Individual Effects (DRIVE) model

## Abstract

Job role ambiguity is becoming more and more common due to the increase in telecommuting caused by the COVID-19 epidemic. In order to understand the internal mechanism of the association between role ambiguity and creativity, this study examined it in the context of the Demands–Resources–Individual Effects (DRIVE) model. Participants were employees from all walks of life in mainland China, with a total of 437 valid data. The results showed that role ambiguity had no significant direct effect on creativity but exerted a negative effect on creativity through the chain mediating effect of affective rumination and perceived stress. A good relationship with a supervisor helped employees reduce their affective rumination when faced with the pressure of role ambiguity. The results show that how employees perceive role ambiguity plays an essential role in determining the potency of the after-effect of role ambiguity. Resources from supervisors can help reduce the negative perception of ambiguous roles.

## 1. Introduction

It has been more than 2 years and COVID-19 is still here. During this period, all parts of the world have experienced intermittent periods of lockdown, which has made telecommuting familiar in specific professional areas and widely implemented in all kinds of nonmanual staff groups. Moreover, the group of telecommuters has also expanded from management and highly paid people to ordinary employees. According to the China Internet Network Information Center (CNNIC, 2021), the number of enterprise WeChat services (a Chinese telecommuting application) grew from 60 million at the end of 2019 to 400 million in December 2020 [1]. Previous research has suggested that telecommuting away from the office may lead to role ambiguity because it does not provide adequate and precise behavioral guidance [2]. Role ambiguity refers to the lack of information related to role expectations, methods for fulfilling general role expectations, and consequences of role performance [3]. In the 1970s and 1980s, some researchers investigated the effects of role ambiguity on job performance and showed inconsistent results. Some studies failed to find a significant relationship between role ambiguity and job performance, while others found a significant negative relationship [3]. With the development of the research on this topic, more and more supporting evidence has been obtained for the negative effect of role ambiguity [2,4,5], with some researchers stating that role ambiguity is purely negative [2]. However, with the increasing emphasis on individual cognitive appraisal and the effect of stress [6], the idea that role ambiguity can also play a positive role began to develop. In a study with 479 participants, Webster and coworkers [7] found that role ambiguity can be evaluated as either obstructive or challenging, and there is no significant difference in the intensity of the two kinds of evaluation. Some studies found that employees would engage in particular behaviors when there is role ambiguity to impress their supervisors [8,9]. In summary, on the basis of the existing studies, there are two possible after-effects of role ambiguity: positive and negative.

As a significant stressor, the effects of role ambiguity on strain and psychological stress have received considerable attention; however, research on role ambiguity and other types of outcomes did not become of interest until 2009 [10]. However, currently, our knowledge about how various types of role stressors affect employee creativity remains fragmented and limited. Indeed, the mechanism linking role stressors and employee creativity has received little attention [11].

Creativity involves generating useful and novel ideas for the birth of new products and services [12]. Employee creativity is essential for organizational innovation, survival, and development [13], especially in today’s complex and uncertain world economic environment. Considerable attention has been paid to organizational management of employees’ creative performance under work pressure [14], but these pressures have mainly focused on time pressure or competition [15]. More attention should be paid to how ambiguous job characteristics and roles across organizations affect employee creativity. Accordingly, this study investigates the relationship between role ambiguity and creativity and explores the internal mechanism connecting them.

### 1.1. Role Ambiguity and Creativity

As mentioned above, role ambiguity can have both positive and negative effects. Role ambiguity refers to the lack of job-related information such as work goals, performance expectations, and job responsibilities that individuals can perceive. If people do not know their responsibilities and what others expect, they will hesitate to take action and feel that they cannot make a difference. Creativity research has also shown that, when distracted by other goals, people automatically resort to habitual behaviors rather than creative ones [10]. Role ambiguity is negatively correlated with creativity, a finding supported by a small number of studies [16,17,18]. However, the positive effects of role ambiguity are also supported. For example, some studies found that a high level of role ambiguity can help employees form a more different understanding and adapt to job roles according to their abilities [19]. When role ambiguity is high, employees’ performance expectations and goals may not be clear. In this case, employees have to speculate and/or set their own goals. Such self-setting challenging goals can demonstrate an individual’s talents and abilities and impress others, including supervisors [9]. Furthermore, according to the research on creativity, an ambiguous context can allow creative action through a process of iteration, trial and error, and self-evaluation [20]. The characteristics of a liberal environment are conducive to high creativity, while people that lack the freedom to make decisions on work tend to show only low creativity. Thus, role ambiguity in an organization can serve as a cradle for developing creative ideas, and creativity can be a logical and intentional natural outgrowth of role ambiguity [10]. In short, role ambiguity and creativity may have both positive and negative associations. However, so far, there is no research on detecting these two paths in a single study. Therefore, this study examines both the positive and the negative effects of role ambiguity on creativity under the same model.

### 1.2. The Demands–Resources–Individual Effects (DRIVE) Model

Existing studies have mainly explained the internal mechanisms underlying the effects of role ambiguity on creativity from the perspectives of destroying intrinsic motivation [21], consuming individual resources [22], or developing negative work attitudes [11,18]. However, these explanatory mechanisms are mainly used to explain the harmful effects of role ambiguity and cannot explain the positive relationship between role ambiguity and creativity. More and more studies related to work stress have begun to emphasize the role of individual cognitive appraisal. The Demands–Resources–Individual Effects (DRIVE) model [6] specifies when job stressors can negatively impact the core of whether the individual perceives stress related to these stressors. That is, perceived stress plays a core mediating role between the stressor and the outcome variables. Therefore, the following hypothesis was tested:

**H1.**  *Perceived stress will mediate the relationship between role ambiguity and creativity*.

The DRIVE model is a systematic but flexible theory. It comprehensively considers the combined effects of various job requirements, job resources, and individual resources on individual health, happiness, work and family balance, and other outcome variables [6]. According to the theory, the key to the negative impact of job demands is whether they make an individual feel stressed. Therefore, it can account for the effect of two opposite outcomes caused by role ambiguity. This study examined the internal mechanism of the connection between role ambiguity and creativity based on the DRIVE model. This involved a systematic investigation of the internal mechanisms and contextual factors that lead to role stressors affecting employee creativity [11].

Although the DRIVE model emphasizes the individual perception of stress between stressors and outcome variables, the theory can be further developed. For example, why do individuals experience or fail to experience strain when facing stressors? According to the DRIVE model, an individual’s subjective assessment is essential in determining the stress response. Therefore, it was predicted that there would be a cognitive, evaluative mediation between role ambiguity and perceived stress. Researchers have recognized that individual cognitive assessment of stressors plays a crucial role in determining stress, but current studies typically ask participants to directly assess the impact of stressors. According to the analysis of existing studies, such direct assessment has significant situational differences. For example, workload, role ambiguity, and role conflict were evaluated as positive or negative in some studies, but there were also situations where they were both positive and negative [23]. Therefore, it is necessary to find a factor that can reflect the individual’s cognitive assessment of the effects of stressors not influenced by the research context.

In recent years, the phenomenon that employees continue to think about their work after work is called work-related rumination (WRR) [24], and this has attracted increased attention. It has two forms of expression, which reflect the individual’s two kinds of assessment of job stressors. This reflection can more truly present the individual’s view on the valence of work stressors and is not easily affected by contextual factors. Affective rumination is a kind of negative cognitive state. The content of affective rumination focuses on the negative emotional experience of work experience [24]. When affective rumination occurs, individuals perceive work as uncontrollable, and there is inevitable stress [25].

Problem-solving pondering reflects an individual’s ongoing psychological examination of a particular problem or evaluation of previous work to see how it could be improved, including thinking in new ways, as well as finding and removing obstacles to developing creative ideas [24]. When engaged in problem-solving pondering, the individual’s goal is to solve the problems encountered in work or promote the development of work [24]. At this time, work is not an uncontrollable pressure [25]. Many studies have confirmed that all kinds of work stressors are the leading causes of work-related rumination [26]. On the basis of the above, it was predicted that work-related rumination would be a mediator linking role ambiguity to perceived stress.

**H2.**  *Role ambiguity will cause perceived job stress through affective rumination*.

**H3.**  *Role ambiguity will reduce perceived job stress through problem-solving pondering*.

A previous study confirmed that affective rumination is significantly negatively correlated with creativity, while problem-solving pondering is significantly positively correlated with creativity [25]. On this basis, the following was predicted:

**H4.**  *Role ambiguity will decrease creativity through affective rumination*.

**H5.**  *Role ambiguity will increase creativity through problem-solving pondering*.

### 1.3. Relationship with Supervisor

According to the DRIVE model, organizational resources can influence the after-effects of work stress. Organizational resources mainly come from the leadership or the policies of the organization. As an organization agent, leaders interact with employees in daily work, directly impacting subordinates’ career success [27]. Especially in China, where the power distance is relatively large, supervisors are closer to employees and exert a more significant influence. Therefore, this study selected the relationship with a supervisor as the representative of organizational resources to analyze its moderating effect on the after-effects of role ambiguity. China is a relationship-oriented society; thus, employees need to establish a good relationship with leaders in an organization [27]. On this basis, it was predicted that a good relationship with the supervisor would alleviate the negative effect of role ambiguity and promote its positive effect as a kind of work resource.

**H6.**  *The relationship with leadership will moderate the relationships between role ambiguity and affective rumination, problem-solving pondering, perceived stress, and creativity*.

**Figure 1:**
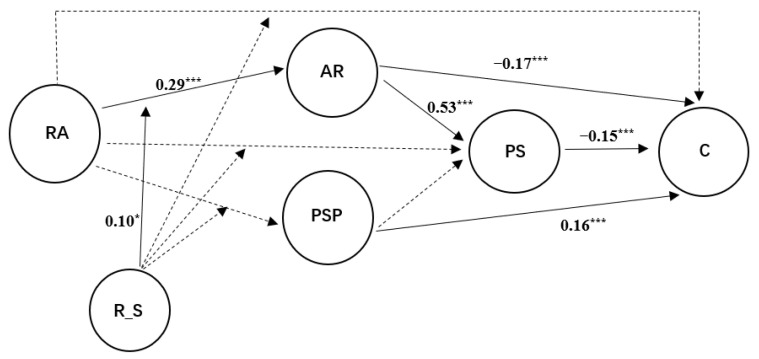
Standardized regression coefficients for the research model. RA = role ambiguity, R_S = relationship with supervisor, AR = affective rumination, PSP = problem-solving pondering, PS = perceived stress, C = creativity; *** *p* < 0.001, * *p* < 0.05. A dotted arrow indicates that the result is not significant. The paths between controlled variables and main variables in the models are not displayed.

The overall research model is shown in Figure 1.

## 2. Methods

### 2.1. Sample and Procedure

Participants were employees from enterprises and public institutions in mainland China, covering 19 provinces. Using a “snowball sampling” methodology, the online questionnaires were sent to the participants via WeChat software 3.7.6 in October 2020. A total of 446 questionnaires were collected, of which nine questionnaires had the phenomenon of selecting the same answer for at least 10 consecutive items, which led to them being deleted. Participation in the study was voluntary and anonymous. After completing the questionnaire, participants received 15 CNY as a thank-you fee.

Of these 437 participants, 210 (48.1%) were male and 227 (51.9%) were female; 240 (54.9%) were in management positions, and 197 (45.1%) were in non-management positions. Their ages ranged from 17 to 64 years (M = 32.22, SD = 6.53), average job tenure was 5.86 years (SD = 0.84), and the average number of working hours per day was 8.61 (SD = 1.96). The participants worked in a range of settings, with 101 (23.2%) in state-owned enterprises, 168 (38.4%) in private enterprises, 119 (27.2%) in public institutions, 17 (3.9%) in governmental agencies, and 32 (7.3%) in other occupational settings. There was a wide range of education levels, with 23 (5.3%) having a high-school education, 64 (14.6%) having a junior college degree, 296 (67.7%) having a bachelor’s degree, and 54 (12.4%) having a master’s degree or above.

All scales were translated into Chinese by means of translation and back-translation. For the disputed areas of translation, a unified scheme was finally developed after discussion among the researchers.

### 2.2. Measures

Work-related rumination. Cropley et al. [28] developed the Work-Related Rumination Questionnaire to measure affective rumination and problem-solving pondering. There are five items in each dimension, scaled from 1 (strongly disagree) to 5 (strongly agree). Example items are as follows:

“Are you troubled by work-related issues when not at work?” (affective rumination).

“In my free time, I find myself re-evaluating something I have done at work.” (problem-solving pondering).

In the current study, Cronbach’s alpha was 0.82 for affective rumination and 0.68 for problem-solving pondering.

Creativity. Employee creativity was measured with a six-item questionnaire developed by Scott and Bruce [29] with responses on a five-point Likert scale from 1 (not at all) to 5 (exceptional degree). The original scale was for rating “others” and was changed into a “self-rating” scale in this study. Doing this may more authentically assess the connection between role ambiguity and creativity. For example, a meta-analysis found that role ambiguity has only a weak association with objective performance and evaluation performance of other leaders or colleagues, but a stronger association with self-evaluation performance [3]. An example item is as follows:

“Please rate on the extent to which yourself searches out new technologies, processes, techniques, and/or product ideas.”

In the current study, Cronbach’s alpha was 0.96.

Other Variables. Role ambiguity (“I feel that I do not understand my role clearly. For example, I am not clear of what is expected of me and what tasks I need to perform.”), relationship with supervisor (“I feel that I get along well with my supervisor. For example, I know where I stand in terms of their opinion of me, my supervisor understands me, and my supervisor recognizes my potential.”), and perceived stress (“Overall, how stressful is your work?”) were measured with three separate items, which were all from the single-item version of the Wellbeing Process Questionnaire (WPQ) [30]. Responses were on a seven-point Likert scale from 1 (not at all) to 7 (very much so). This scale’s good reliability and validity have been strongly supported in many studies [31].

Control Variables. On the basis of results from prior research [32], age, gender, working tenure, working setting, education level, average working hours per day, and management position were used as the control variables.

### 2.3. Assessment of Common Method Variance

The data in the present study were collected via self-administered questionnaires. Therefore, common method variance could inflate the strength of observed relationships [33]. Two methods were used to reduce the impact of common method variance. First, Harman’s single-factor test was used to determine whether each measure explained unique variance in the data. Exploratory factor analysis showed that there were three factors (λ > 1) when there was no rotation. The first factor explained 34.02% of the variance, which could not account for most covariance measures. Second, confirmatory factor analysis was performed on the data. The results showed that the fitting degree of the single-factor model (χ^2^/df = 7.84, CFI = 0.76, TLI = 0.73, GFI = 0.70, RMSEA = 0.13) was significantly lower than that of the six-factor model (χ^2^/df = 2.23, CFI = 0.96, TLI = 0.95, GFI = 0.93, RMSEA = 0.05).

## 3. Results

### 3.1. Preliminary Analyses

Table 1 presents the means, standard deviations, and correlations. The results showed that role ambiguity, affective rumination, and perceived stress were significantly negatively correlated with creativity. Role ambiguity was positively correlated with affective rumination and was not significantly associated with problem-solving pondering. Problem-solving pondering was positively correlated with creativity. Role ambiguity and affective rumination were positively correlated with perceived stress; problem-solving pondering was not significantly associated with perceived stress. Relationship with the supervisor was positively correlated with problem-solving pondering and creativity, but negatively correlated with affective rumination and perceived stress. This provided some preliminary support for hypotheses H1 to H6.

### 3.2. Hypothesis Testing

**Figure 2:**
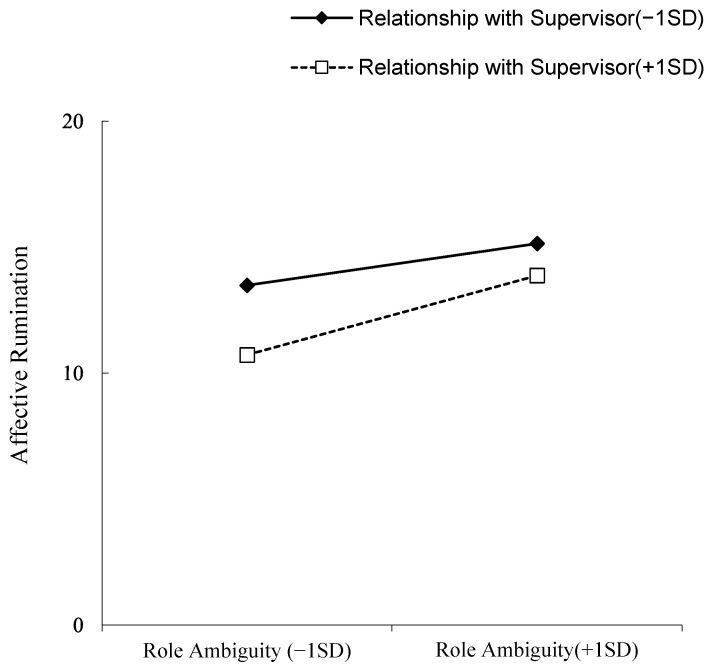
Simple slope effect diagram.

The model was tested with AMOS21. The overall model was a good fit (χ^2^/df = 2.27, CFI = 0.92, IFI = 0.92, GFI = 0.96, RMSEA = 0.05). Statistical results showed no significant correlation between role ambiguity and creativity (β = −0.63, *p* = 0.174). Role ambiguity was not significantly associated with perceived stress (β = −0.02, *p* = 0.643). Perceived stress was negatively associated with creativity (β = −0.15, *p* < 0.001). Hypothesis 1 was not supported. Role ambiguity was positively associated with affective rumination (β = 0.29, *p* < 0.001), but showed no significant correlation with problem-solving pondering (β = −0.01, *p* = 0.776). Affective rumination was positively associated with perceived stress (β = 0.53, *p* < 0.001) and negatively associated with creativity (β = −0.17, *p* < 0.001). On the other hand, problem-solving pondering was not significantly associated with perceived stress (β = 0.07, *p* = 0.096) and positively associated with creativity (β = 0.16, *p* < 0.001). Hypotheses H2 and H4 were supported, while hypotheses H3 and H5 were not supported. Therefore, affective rumination and perceived stress play a chain mediating role between role ambiguity and creativity, and the results are summarized in Table 2. In order to analyze the indirect effect and 95% confidence interval (CI) of the mediation model, the PROCESS macro for SPSS developed by Hayes (2013) was used to perform follow-up analyses. Table 3 shows the results. The product of role ambiguity and relationship with a supervisor only significantly positively predicted the relationship between role ambiguity and affective rumination (β = 0.10, *p* < 0.05), indicating that the relationship with the supervisor could significantly moderate role ambiguity-induced affective rumination. Simple slope analysis was further carried out, and the results are shown in Figure 2. As shown in Figure 2, when employees have a good relationship with their supervisors, there is less affective rumination due to role ambiguity pressure.

## 4. Discussion

The present results suggest that role ambiguity is not directly associated with creativity but negatively affects creativity through a partial mediation of affective rumination. In addition, role ambiguity did not directly affect strain, but indirectly induced strain through affective rumination. These findings show that the affective rumination of employees is the key to the negative effect of role ambiguity. The phenomenon that the direct effect of role ambiguity is not significant has been supported by some studies. For example, role ambiguity has no significant direct relationship with workplace harassment [34] or job performance [35]. These results suggest that, when analyzing the effect of role ambiguity, one may be faced with more complicated situations and should not simply and directly presume role ambiguity as a significant negative effect. Moreover, there was a finding that the direct effect of role ambiguity is not significant or even nonlinear [36].

According to our results, when faced with role pressure, such as ambiguous job role responsibilities and job role requirements, employees will have negative work-related rumination, which will make employees experience perceived stress from the ambiguous job role, thus reducing their creative performance at work. Therefore, it is of great practical significance to find boundary conditions that can help employees reduce their affective rumination in the face of role ambiguity. In response to this question, this study further analyzed the moderating role of the relationship with a supervisor. A good relationship with a supervisor means a good leader–member exchange. A large number of studies have confirmed that such a good relationship can bring many positive resources to employees, such as helping employees get more support from their leaders, increasing their emotional commitment and job satisfaction [37]. Consistent with these results, this study confirmed that a good relationship with a supervisor could alleviate negative work rumination in the face of role ambiguity.

This study failed to find a mediating role of problem-solving pondering between role ambiguity and creativity. This is mainly because there was no significant link between role ambiguity and problem-solving pondering. Research has found that intrusion by work roles in non-work roles (responding to work-related emails and messages from home) only leads to problem-solving rumination, not affective rumination [26]. This suggests that different types of role stress pairs may correspond to different types of work-related rumination. For the role pressure within their control range (e.g., work interferes with life), employees tend to think in a problem-solving way, while, for the role pressure outside their control range, such as role ambiguity, employees tend to carry out affective rumination. This point is consistent with the view that the controllability of work stress is the key reason for which type of work-related rumination occurs [24,38]. The unclear responsibility requirements, vague role-performance expectations, and other role ambiguous pressures are at the organizational level, which is difficult to solve by relying on individual employees’ cognitions. Therefore, in the face of role ambiguity, employees will not think about it repeatedly to solve problems.

This study attempted to analyze the possible positive and negative effects of role ambiguity on creativity in a single model. Only the negative path was supported. However, this does not mean that the positive effect of role ambiguity on creativity does not exist. O’Connor et al. (2022) directly separated hindrance and challenging role ambiguity. This definition, it turns out, is necessary to analyze when a leader’s tolerance of ambiguity benefits subordinates’ performance [39]. Perhaps this approach can be used to analyze the positive effects of role ambiguity in the future.

In conclusion, this study provided further evidence to support the idea that individual cognition plays a central role in determining the impact of stressors, which may provide a reasonable way to explain the double-edged sword effect of role ambiguity found in previous studies. When employees interpret ambiguous role requirements as uncontrollable adverse events, they will experience pressure and adopt conservative and noninnovative working methods. However, a good relationship between employees and supervisors can alleviate employees’ negative interpretation of ambiguous roles.

## 5. Conclusions

### 5.1. Contributions

Firstly, in response to Wang et al. [11], this study analyzed the association between role ambiguity and employees’ creativity, which provides more information on this topic. Using the DRIVE model, the internal mechanism of role ambiguity affecting work creativity was explored. It was clear that role ambiguity itself had no significant effect on creativity but harmed creativity through the chain mediating effect of affective rumination and perceived stress.

In addition, this study further extended the DRIVE model to show that there is a cognitive assessment process between stressors and perceived stress, namely, work-related rumination. This process determines whether employees feel stressed when exposed to stressors. Therefore, employees’ rumination about work stress should be considered when applying the model to explain the mechanism of stressors’ action in future studies.

Lastly, previous studies have found that role ambiguity has both negative and positive effects, but no study has tested the double-edged sword effect of role ambiguity in the same model. Using the DRIVE model, this study analyzed the two approaches to role ambiguity simultaneously for the first time. Although only the adverse effects of role ambiguity were identified, this study suggests that researchers concerned with role ambiguity should adopt such a comprehensive model instead of a predetermined negative or positive perspective when analyzing the after-effects of role ambiguity.

### 5.2. Practical Implications

On the basis of the results of this study, we suggest that organizational managers should pay attention to establishing precise job role requirements and responsibility scope for employees, especially for telecommuting employees, when formulating various work policies and systems. This has at least two advantages; one is to directly reduce employees’ negative rumination on the job, i.e., affective rumination, which has a wide range of adverse effects on employees’ health [28], wellbeing [40], and job performance [25]. The other is to indirectly avoid the reduction of employees’ creativity under vague role requirements.

This study suggests that, if organizational managers want to improve employees’ creativity by setting work challenges, they need to be cautious, at least not by not explicitly proposing responsibility requirements and performance expectations. Because such job role requirements are not specific enough, if it is not implemented well, it will only make employees experience pressure and lose motivation, which will damage employees’ creativity.

This study found a meaningful way to alleviate the harmful effects of role ambiguity, namely, a good relationship with leaders. This indicates that the negative impact of stressors caused by organizational systems or policies on employees can be avoided by humanistic care from supervisors and other leaders. This suggests that the organization’s management should provide more support resources for employees in daily work to offset the adverse effects of unreasonable organizational policies. It also emphasizes that employees should establish a good working relationship with their leaders, especially supervisors.

### 5.3. Limitations and Future Research

Despite the unique contribution of this study, some limitations need to be addressed. First of all, in terms of research methods, cross-sectional research can effectively verify the research model, but it cannot make direct causal inferences. Future studies should take time into account, using longitudinal or daily measurements to understand better causality and the stability of the model across time. Furthermore, to better control for the effects of common method bias, in addition to what was achieved in this study, data can also be collected from extra sources, such as measuring employee creativity through managers.

Secondly, this study only analyzed the stress-buffering effect of one kind of work resource. Future research could expand the types of resources (such as information resources) or select more direct resource representatives (such as supervisor support) to explore more ways to reduce the negative effect of role ambiguity.

Thirdly, the DRIVE model comprehensively considers the effects of various types of stressors, work resources, and individual differences. Although this study developed the DRIVE model, these other factors were not considered. The moderating effect of individual differences was not addressed here. In future studies on the relationship between role ambiguity and creativity, individual differences should be taken into account in order to make full use of the DRIVE model to obtain a more comprehensive understanding of the relationship between role ambiguity and creativity.

Lastly, this study failed to find a direct and significant link between role ambiguity and problem-solving pondering. This suggests that specific external conditions may be required to establish a positive connection between role ambiguity and problem-solving pondering. As Martínez-Díaz and coworkers [19] found, the positive effect of role ambiguity on job engagement is achieved under the moderation of performance recognition. Therefore, future research should explore the boundary conditions of the connection between role ambiguity and positive work-related rumination to identify additional possible positive effects of role ambiguity.

## Figures and Tables

**Table 1:** Descriptive statistics and correlations among study variables.

Variables	1	2	3	4	5	6
1 RA	1					
2 AR	0.34 **	1				
3 PSP	−0.05	0.03	1			
4 R_S	−0.16 **	−0.30 **	0.17 **	1		
5 PS	0.18 **	0.56 **	0.06	−0.25 **	1	
6 C	−0.26 **	−0.38 **	0.22 **	0.47 **	−0.29 **	1
*M*	2.80	13.24	16.94	5.20	3.97	28.13
*SD*	1.65	4.07	2.78	1.15	1.39	7.61

N = 437. Note: RA = role ambiguity, R_S = relationship with supervisor, AR = affective rumination, PSP = problem-solving pondering, PS = perceived stress, C = creativity; ** *p* < 0.01.

**Table 2:** Results of multiple regression analyses with creativity as the dependent variable.

Variable	AR		PSP		Perceived Stress		Creativity	
	β	SE	β	SE	β	SE	β	SE
RA	0.29 ***	0.18	−0.01	0.13	−0.02	0.06	−0.63	3.37
AR					0.53 ***	0.02	−0.17 ***	0.08
PSP					0.07	0.02	0.16 ***	0.09
Perceived stress							−0.15 ***	0.22
R_S × RA	0.10 *	0.18	−0.12 *	0.13	0.06	0.06	4.69	27.50
Age							−0.30 ***	0.05
Gender							−0.03	0.51
Education							−0.01	0.39
Working tenure							0.02	0.37
Working setting							−0.23 ***	0.23
Management position							−0.23 ***	0.51
Average working hours per day							0.00	0.13

N = 437. β = standardized coefficients. RA = role ambiguity, R_S = relationship with supervisor, AR = affective rumination, PSP = problem-solving pondering; * p < 0.05; *** p < 0.001.

**Table 3:** Bootstrapping indirect effect and 95% confidence interval (CI) for the mediation model.

	IEV	Boot SE	95% CI Limit	
			(Lower)	(Upper)
RA → AR → C	−0.33	0.09	−0.55	−0.18
RA → PS → C	0.01	0.04	−0.06	0.08
RA → AR → PS → C	−0.15	0.05	−0.27	−0.07

N = 437. The results are based on 1000 bootstrap samples. IEV = indirect effect value of RA on C, CI = confidence interval, RA = role ambiguity, AR = affective rumination, PS = perceived stress, C = creativity.

## Data Availability

The data presented in this study are available on request from the corresponding author. The data are not publicly available due to privacy policy restrictions.
